# Survival of Naïve T Cells Requires the Expression of Let-7 miRNAs

**DOI:** 10.3389/fimmu.2019.00955

**Published:** 2019-05-03

**Authors:** Elena L. Pobezinskaya, Alexandria C. Wells, Constance C. Angelou, Eric Fagerberg, Esengul Aral, Elizabeth Iverson, Motoko Y. Kimura, Leonid A. Pobezinsky

**Affiliations:** ^1^Department of Veterinary and Animal Sciences, University of Massachusetts, Amherst, MA, United States; ^2^Department of Immunology, Graduate School of Medicine, Chiba University, Chiba, Japan

**Keywords:** CD4, CD8, peripheral homeostasis, post-transcriptional, DICER, apoptosis, mitochondria, bcl-2

## Abstract

Maintaining the diversity and constant numbers of naïve T cells throughout the organism's lifetime is necessary for efficient immune responses. Naïve T cell homeostasis, which consists of prolonged survival, occasional proliferation and enforcement of quiescence, is tightly regulated by multiple signaling pathways which are in turn controlled by various transcription factors. However, full understanding of the molecular mechanisms underlying the maintenance of the peripheral T cell pool has not been achieved. In the present study, we demonstrate that T cell-specific deficiency in let-7 miRNAs results in peripheral T cell lymphopenia resembling that of *Dicer1* knockout mice. Deletion of let-7 leads to profound T cell apoptosis while overexpression prevents it. We further show that in the absence of let-7, T cells cannot sustain optimal levels of the pro-survival factor Bcl2 in spite of the intact IL-7 signaling, and re-expression of Bcl2 in let-7 deficient T cells completely rescues the survival defect. Thus, we have uncovered a novel let-7-dependent mechanism of post-transcriptional regulation of naïve T cell survival *in vivo*.

## Introduction

Upon maturation in the thymus, naïve T cells populate the peripheral lymphoid organs. Since thymic output decreases with age due to thymus involution, it is important that the naïve T cell pool is maintained in the periphery for the lifespan of the organism. Thus, maintenance of peripheral T cells is a tightly regulated process that first and foremost ensures the prolonged survival of T cells and, secondly, provides signals that keep naïve T cells in a quiescent state, thus preventing non-specific activation.

Various signaling pathways are involved in the maintenance of the peripheral T cell pool. It has been well-established that a combination of homeostatic signals, such as low-affinity T cell receptor (TCR) interactions with self-peptide-MHC complexes and common gamma-chain receptor cytokines, especially IL-7, are indispensable for the prolonged survival of peripheral T cells (1–3). In fact, arginine methyltransferase PRMT5 has been recently shown to support T cell maintenance by regulating strength of signaling through common gamma-chain receptors (4). Constant recirculation of naïve T cells through secondary lymphoid organs appears to be important for them to receive necessary homeostatic signals (5). Interaction between chemokine CCL19 and its receptor CCR7, as well as S1P signaling have been also demonstrated to support survival of naïve T cells (6, 7). In addition, control of metabolic signaling pathways is essential for T cell maintenance since deficiency in tuberous sclerosis complex 1, that restrains mTOR activity, leads to both peripheral T cell lymphopenia and loss of quiescence (8–10). Keeping cellular stress at low levels also appears to be important for T cell homeostasis, as mutation in *Kdelr1* gene, a negative regulator of the stress response, results in increased apoptosis of T cells (7) and Schlafen2 deficiency, results in chronic ER stress and compromised T cell quiescence (11).

Transcriptional control of T cell homeostasis has been extensively studied. Forkhead box family transcription factors have been shown to play an essential role in the regulation of T cell maintenance. For example, Foxo1 is necessary for the survival of naïve T cells (12–14), while FoxJ1 (15) and FoxP1 (16, 17) reinforce the quiescent state. Ets1 and GABP, both members of the Ets transcription factor family, were also implicated in naïve T cell homeostasis (18–20). Post-transcriptional control of T cell maintenance, however, remains largely unknown. RNA interference (RNAi) is the primary mechanism responsible for global post-transcriptional regulation of gene expression in multiple biological processes. RNAi is mediated primarily by microRNAs (miRNAs), short non-coding RNAs, that repress protein synthesis mostly by destabilizing target mRNAs in a sequence-specific manner (21, 22). Dicer is one of the essential enzymes involved in miRNA biogenesis (23) and, as RNAi is indispensable for mouse development, *Dicer1* knockout mice are embryonically lethal (24). Mice with a T cell-specific deletion of Dicer demonstrate a dramatic reduction in thymocyte numbers and dysregulated differentiation of CD4^+^ and NKT cells (25, 26). Importantly, it has been observed that the frequencies of peripheral T cell subsets in Dicer-deficient animals are severely reduced, suggesting that miRNAs may also be important for peripheral T cell homeostasis (26). With the exception of one report that demonstrates the role of miR-191 in supporting T cell survival (27), specific miRNAs and the mechanism by which they control T cell maintenance are not known.

We have recently shown that high levels of let-7 miRNAs expressed in naïve T cells are important for the maintenance of the quiescent state (28). In this study, we further explored the role of let-7 in peripheral T cell homeostasis. We show that similar to Dicer-deficient mice, let-7-deficient animals develop severe peripheral T cell lymphopenia which appears to be a result of impaired survival due to the low expression of the pro-survival factor Bcl2. In addition, we demonstrate that let-7 controls Bcl2-mediated survival through an IL-7-independent mechanism.

## Results

### Peripheral T Cell Lymphopenia in Dicer-Deficient and Lin28Tg Mice

**Figure 1:**
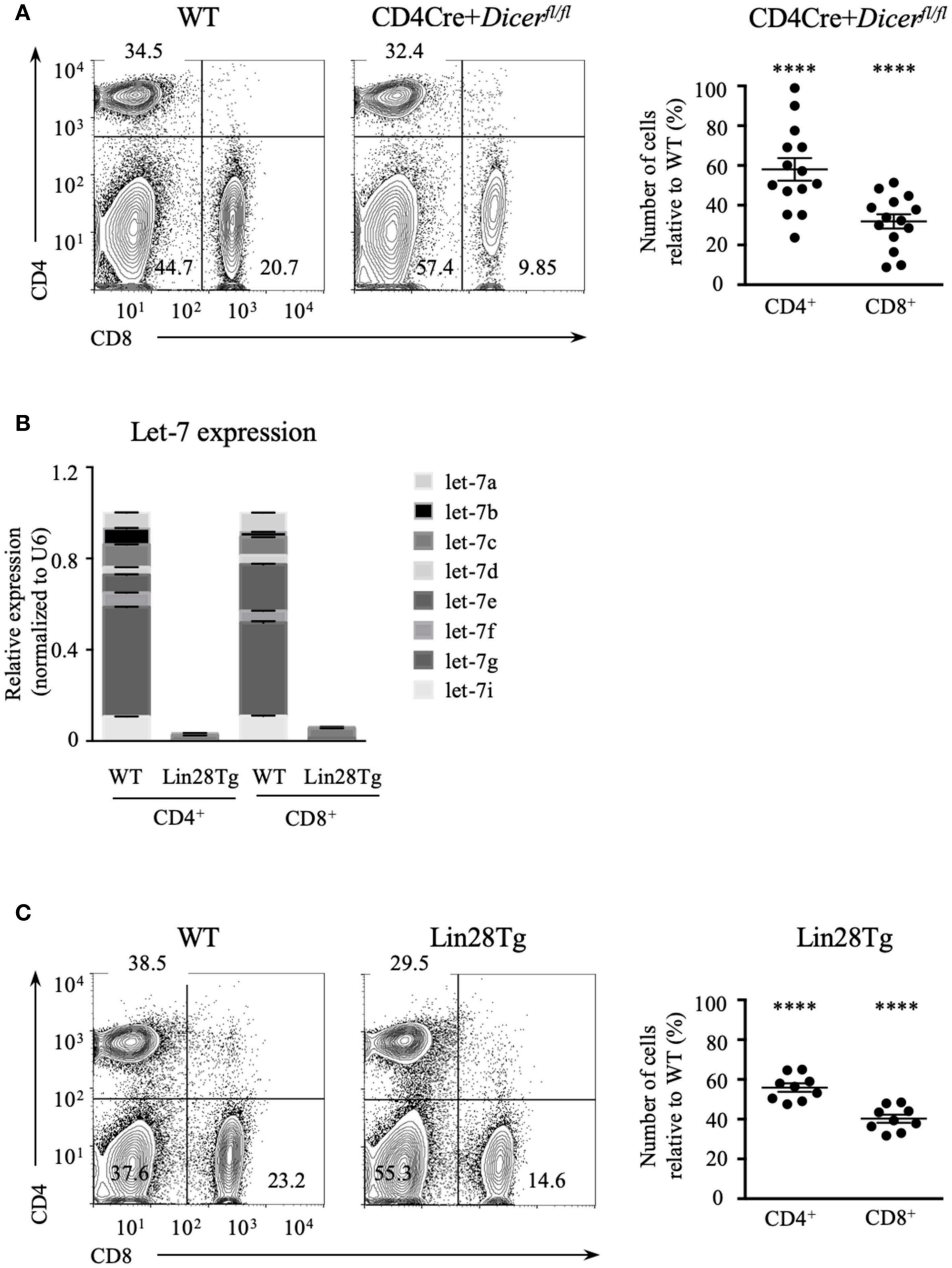
Let-7 is necessary to maintain peripheral T cell numbers and frequency. **(A)** CD4 and CD8 expression on lymph node cells from wild type and CD4Cre^+^*Dicer*^*fl*/*fl*^ mice (left). Number of CD4 and CD8 lymph node T cells in CD4Cre^+^*Dicer*^*fl*/*fl*^ mice normalized to wild type littermate controls (right). **(B)** Quantitative RT-PCR analysis of individual let-7 miRNAs in CD4 and CD8 lymph node T cells from wild type and Lin28Tg mice. **(C)** CD4 and CD8 expression on lymph node cells from wild type and Lin28Tg mice (left). Number of CD4 and CD8 lymph node T cells in Lin28Tg mice normalized to wild type littermate controls (right). Data are from at least three independent staining experiments and are displayed as mean ± SEM of each population from 14 **(A)** and 9 **(B)** individual mice, ****p < 0.0001.

Dicer ablation in T cells results in the reduction of mature CD4^+^ and CD8^+^ lymphocytes (26) suggesting that RNA interference may have a role in their maintenance. We confirmed this result by analyzing the abundance of T cells using CD4Cre^+^*Dicer*^*fl*/*fl*^ mice (Figure 1A). The total numbers of CD4^+^ and especially CD8^+^ T cell populations in the lymph nodes (LNs) were significantly lower in Dicer-deficient animals in comparison to wild type littermate controls, thus demonstrating that T cell-specific deletion of Dicer leads to T cell lymphopenia in the periphery. To address the question of which particular miRNAs are involved in the control of T cell homeostasis, we focused on the largest family of miRNAs, *lethal*-7 (let-7). Let-7 is a family of 12 miRNA paralogs in mammals, is expressed in various tissues and is highly conserved throughout evolution (29). Importantly, abundant expression of let-7 miRNAs in naïve T cells is essential to maintain quiescence (28). Altogether, this makes the let-7 family of miRNAs a strong candidate for controlling T cell homeostasis.

To test whether let-7 miRNA expression is needed for T cell homeostasis, and whether the absence of these miRNAs can account for T cell lymphopenia in mice, we took advantage of T-cell specific *lin28b* transgenic (Lin28Tg) animals (30). Lin28B, one of the two Lin28 embryonic proteins, is known to inhibit let-7 biogenesis by specifically binding to a loop region of let-7 precursors, targeting them for degradation (31–33); therefore, Lin28Tg T cells do not express mature let-7 miRNAs (Figure 1B). Lin28Tg mice demonstrated a decrease in the frequency of CD4^+^ and particularly CD8^+^ mature lymph node T cells in comparison to wild type littermate controls, while also exhibiting a profound reduction in the total numbers of CD4^+^ and CD8^+^ T cells (Figure 1C). Thus, in line with the published results (34), our data indicate that Lin28Tg animals develop severe T cell lymphopenia, similar to mice with T cell-specific ablation of Dicer.

### Impaired Survival of Let-7-Deficient T Cells

The reduced number of T cells in the periphery of Lin28Tg mice could be a result of a developmental defect or impaired peripheral homeostasis. We have shown previously that both thymocyte numbers and frequencies of thymic populations are unchanged in Lin28Tg mice when compared to wild type controls (30), which suggests that T cell development is uncompromised, pointing toward a potential defect in the maintenance of T cells in the periphery. Since naïve T cells predominantly receive survival signals and divide very rarely (3), it is unlikely that impaired homeostatic proliferation would fully account for lymphopenia in Lin28Tg mice. In fact, we have previously demonstrated, and confirmed here again that the proliferation of Lin28Tg T cells is even higher than that of wild type T cells, as shown by increased BrdU incorporation *in vivo* [(28) and Figure S1]. Thus, proliferation of T cells is not suppressed in the absence of let-7.

**Figure 2:**
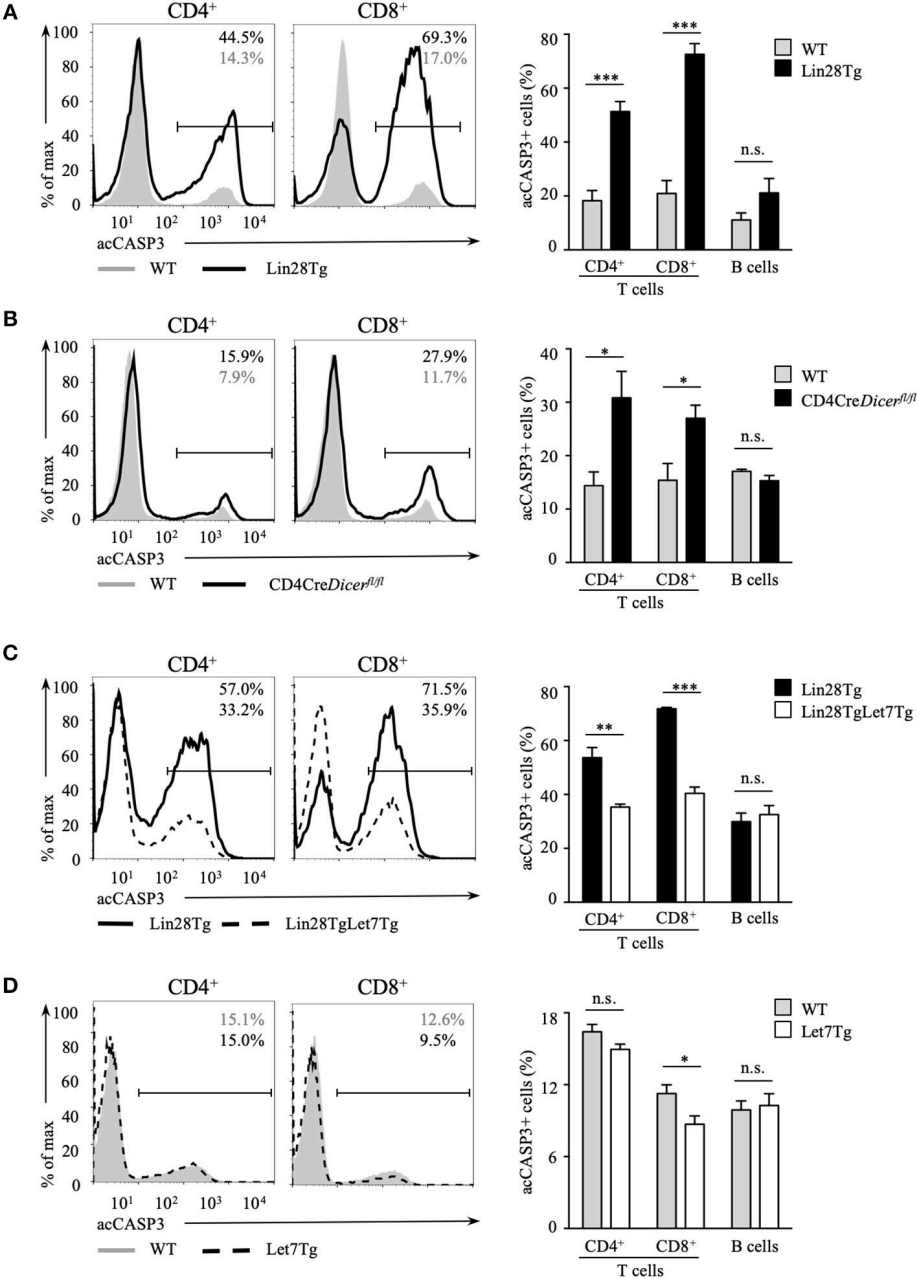
Let-7 is essential in protecting T cells from apoptosis. Intracellular staining for active Caspase-3 (left) and frequency of acCasp3^+^ population (right) in CD4 and CD8 lymph node T cells from **(A)** wild type (*n* = 4) and Lin28Tg (*n* = 4) mice, **(B)** wild type (*n* = 6) and CD4Cre^+^*Dicer*^*fl*/*fl*^ (*n* = 6) mice, **(C)** Lin28Tg (*n* = 3) and Lin28TgLet7Tg (*n* = 3) mice, and **(D)** wild type (*n* = 4) and Let7Tg (*n* = 4) mice, cultured for 18 h in media. Data are representative of at least three independent staining experiments and are displayed as mean ± SEM of each population from all mice; n.s., not significant (*p* > 0.05), *p < 0.05, **p < 0.01, ***p < 0.001.

Next, we tested whether the survival of let-7-deficient T cells is affected. Since it is challenging to detect apoptotic cells *in vivo* because of their rapid clearance in the organism, we set up an *in vitro* assay to evaluate apoptosis. Lin28Tg T cells were cultured without stimulation in growth media only and then assessed for the presence of the active form of caspase-3 (acCasp3) as an indication of on-going apoptosis. Indeed, profound cell death was observed in the T cells of Lin28Tg mice. Both CD4^+^ and CD8^+^ let-7-deficient T cells demonstrated significantly higher frequencies of acCasp3-positive cells in comparison to their wild type littermate controls (Figure 2A), with CD8^+^T cells showing a more substantial difference. B cells from Lin28Tg and wild type mice were analyzed as a negative control and, as expected, did not have any difference in cell death. Interestingly, CD4-Cre^+^*Dicer*^fl/fl^ T cells were also more susceptible to apoptosis (Figure 2B), suggesting a similar mechanism of cell death in dicer-deficient T cells. We noticed that dicer-deficient T cells died less than Lin28Tg cells perhaps due to some unknown effects that are mediated by the absence of other miRNAs in these lymphocytes. These data strongly demonstrate that Lin28Tg T cells have a survival defect due to increased apoptosis.

To confirm that compromised survival is let-7-dependent and is not due to some unknown Lin28-mediated function, we generated double transgenic mice where doxycycline-inducible let-7g miRNA, one of the let-7 family members, was re-expressed in Lin28Tg mice. Since all mature miRNAs from the same family have identical seed sequence and will target the same mRNAs, it is reasonable to assume that evaluating the effects of only let-7g miRNA is sufficient to say that other let-7 miRNAs have similar functions. Note that the let-7g miRNA transgene encodes a chimeric molecule with the loop region that cannot be recognized by Lin28 and thus transgenic let-7g can be expressed even in the presence of Lin28 (35). Lin28TgLet7Tg T cells were then analyzed for caspase-3 activation in an *in vitro* assay. The cell death was largely rescued in T cells with forced let-7 expression, as the frequency of acCasp3 positive Lin28TgLet7Tg T cells was significantly reduced when compared to Lin28Tg T cells (Figure 2C). Moreover, even Let7Tg T cells demonstrated improved survival in comparison to control wild type T cells (Figure 2D) and, again, a larger effect was observed in CD8^+^ T cells. Altogether, our results demonstrate that let-7 is essential for the survival of naïve T cells in the periphery.

### Let-7 Controls T Cell Survival in a Cell-Intrinsic Manner

**Figure 3:**
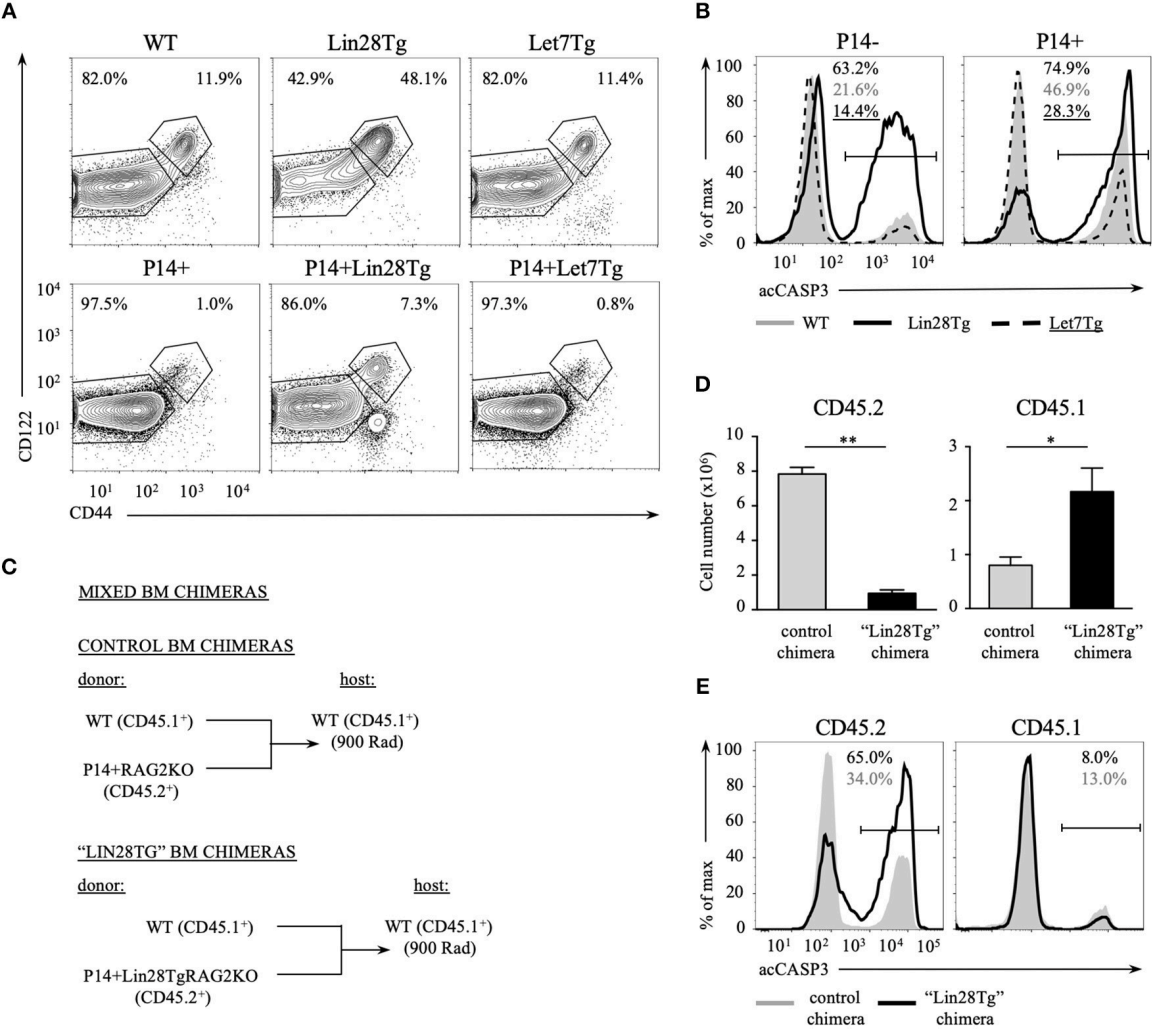
Impaired survival of let-7-deficient T cells is cell-intrinsic. **(A)** Surface expression of CD44 and CD122 memory markers on CD8 T cells from lymph nodes of polyclonal (top) and P14+Rag2KO (bottom) wild type, Lin28Tg and Let7Tg mice. **(B)** Intracellular staining for active Caspase-3 in CD8 lymph node T cells from polyclonal (left) and P14^+^ Rag2KO wild type, Lin28Tg and Let7Tg mice (right) cultured for 12 h in media at 37°C. **(C)** Schematic representation of the generation of mixed bone marrow chimeras. **(D)** Total number of CD45.2^+^ and CD45.1^+^ CD8 lymph node T cells recovered from control (*n* = 3) and “Lin28Tg” (*n* = 3) mixed BM chimeras. Data are displayed as mean ± SEM, *p < 0.05, **p < 0.01. **(E)** Intracellular staining for active Caspase-3 in the same populations of CD8 lymph node T cells cultured for 18 h in media. Data in **(A,B,E)** are representative of three independent staining experiments.

Impaired survival of let-7-deficient T cells could be a bystander effect determined by environmental cues. This possibility is especially relevant in the case of Lin28Tg animals. In healthy wild type mice the majority of peripheral T cells are naïve and only 10–20% of all T cells possess a CD44^hi^CD122^hi^ memory-like phenotype (36). We have previously shown that a very high percentage of Lin28Tg T cells have a memory-like phenotype due to the overproduction of IL-4 by a subpopulation of PLZF^hi^ NKT cells that are overrepresented in these mice (30). Indeed, while the majority of wild type T cells (82%) were naïve (CD44^low^CD122^low^), almost a half of Lin28Tg T cells were CD44^high^CD122^high^ (Figure 3A, upper panel). To exclude the influence from IL-4 and other factors potentially produced in Lin28Tg mice on the survival of T cells, we generated P14^+^Lin28Tg mice on a Rag2KO background, where P14 is a T cell receptor specific to the lymphocytic choriomeningitis virus peptide gp33-41. The naïve phenotype of T cells was rescued in P14^+^Lin28TgRag2KO animals almost to the wild type level (Figure 3A, lower panel). More importantly, the survival of P14^+^Lin28TgRag2KO T cells still remained compromised, while P14^+^Let7TgRag2KO T cells survived better than P14^+^Rag2KO control T cells (Figure 3B). This experiment demonstrates that bystander effects mediated by other T cell subsets are not responsible for the increased apoptosis of let-7-deficient T cells.

To test whether lymphopenia and impaired survival of Lin28Tg T cells are cell-intrinsic, we generated mixed bone marrow (BM) chimeras, where lethally irradiated C57BL/6 host mice (CD45.1^+^) were reconstituted either with a mixture of congenically marked BM cells from wild type (CD45.1^+^) and P14^+^Lin28TgRag2KO (CD45.2^+^) mice to make “Lin28Tg” BM chimera or with a mixture of BM cells from wild type (CD45.1^+^) and P14^+^Rag2KO (CD45.2^+^) mice for control BM chimeras (Figure 3C). First, we assessed the number of CD8^+^ T cells recovered from the lymph nodes of chimeric mice 8 weeks post-reconstitution. Analysis of the CD45.2^+^ population revealed a marked reduction in P14^+^Lin28TgRag2KO T cells in the lymph nodes of “Lin28Tg” BM chimeras in comparison to P14^+^Rag2KO T cells from control BM chimeric mice (Figure 3D). Conversely, the population of CD45.1^+^ wild type cells in “Lin28Tg” chimeras was overrepresented, which can be attributed to the compensatory expansion of wild type cells in an attempt to fill the T cell niche. Next, the LN cells from control and “Lin28Tg” BM chimeras were analyzed for the resistance to apoptosis *in vitro*. P14^+^Lin28TgRag2KO (CD45.2^+^) T cells from “Lin28Tg” BM chimera had a much higher proportion of acCasp3-positive cells in comparison to P14^+^Rag2KO (CD45.2^+^) T cells from control BM chimera (Figure 3E). Thus, the lymphopenia observed in Lin28Tg mice as a result of the survival defect of let-7-deficient T cells is cell-intrinsic. This conclusion was further confirmed in an adoptive transfer experiment where equal numbers of mature lymph node wild type and Lin28Tg T cells were mixed and transferred into Rag2KO host mice and analyzed at different times starting 1 week after transfer (Figure S2). Wild type T cells were able to repopulate lymph nodes of Rag2KO mice and outcompete let-7-deficient T cells. Collectively, these data demonstrate that the impaired homeostasis of Lin28Tg T cells is due to increased apoptosis, which appears to be a cell-intrinsic property of let-7-deficient T cells.

### Suboptimal Bcl2 Levels Lead to Apoptosis in Let-7-Deficient T Cells

There are two pathways that can lead to the activation of caspase 3 and thus apoptosis: extrinsic and intrinsic (37). We sought to determine which mechanisms are responsible for the survival defect of let-7-deficient naive T cells. The extrinsic pathway of apoptosis is induced by ligation of death receptors belonging to the tumor necrosis factor receptor superfamily (Tnfrsf) that consists of eight members, three of which are known to induce apoptosis in T cells: Tnfrsf10b (DR5), Tnfrsf1a (Tnfr1) and Fas (38, 39). We evaluated expression of all three receptors. Neither of the receptors nor their ligands, *Tnfsf10* (TRAIL) and *Fasl*, demonstrated any significant difference in expression on the mRNA level between wild type and Lin28Tg T cells (Figures S3A–C). However, *Fas* mRNA was slightly elevated in Lin28Tg T cells, which prompted us to analyze surface expression of the protein. Fas protein expression appeared to be significantly increased in Lin28Tg T cells when compared to wild type control (Figure S3D). There are several reports demonstrating that the let-7 miRNA family can target Fas and Fasl (40–42). Moreover, Fas has been shown to regulate the homeostasis of peripheral T cells (43). To test whether apoptosis of let-7-deficient T cells is mediated by Fas, Lin28Tg mice were crossed with Fas-deficient mice (Fas^lpr/lpr^) and *in vitro* survival of T cells from Lin28Tg mice and Fas-deficient Lin28Tg mice was compared. Surprisingly, Fas deficiency did not rescue the impaired survival of Lin28Tg T cells, thus excluding the possibility of Fas-induced T cell death in the absence of let-7 miRNAs (Figure S3E). Altogether, the data indicate that death receptor-mediated apoptosis is unlikely to be involved in the impaired survival of let-7-deficient T cells.

**Figure 4:**
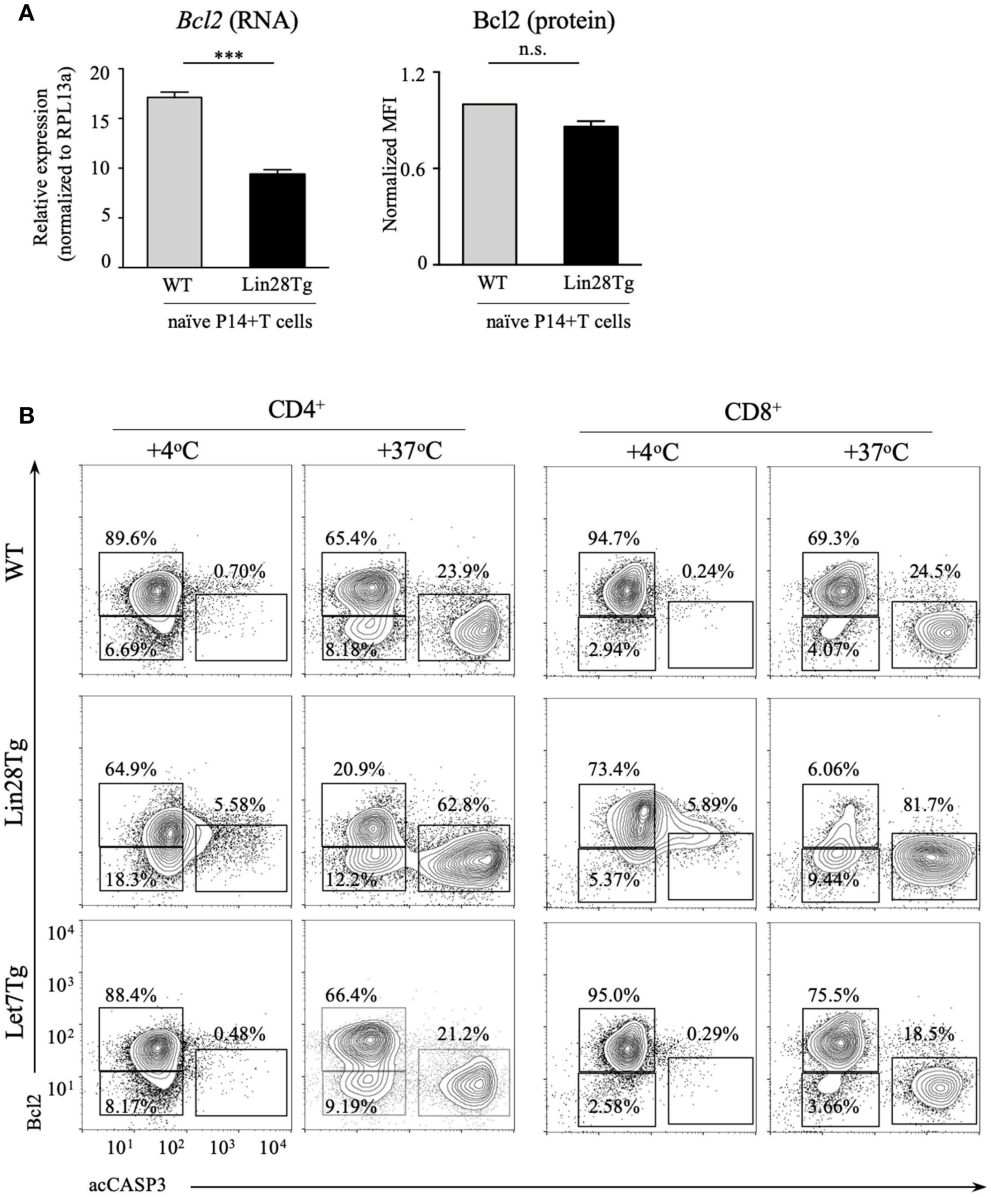
Bcl2 expression is reduced in let-7 deficient T cells. **(A)** Quantitative RT-PCR analysis of *Bcl2* expression, presented relative to expression of the ribosomal protein *Rpl13a* (left) and MFI of Bcl2 normalized to wild type (right) in CD8 T cells from lymph nodes of P14+Rag2KO wild type and P14+Lin28TgRag2KO mice. Data are displayed as mean ± SEM of technical triplicates **(B)** Intracellular staining for Bcl2 and active Caspase-3 in CD4 and CD8 lymph node T cells from wild type, Lin28Tg and Let7Tg mice, cultured for 18 h in media. Data are representative of three independent experiments **(A)** and two **(A)** and three **(B)** independent staining experiments; n.s., not significant (*p* > 0.05), ***p < 0.001.

**Figure 5:**
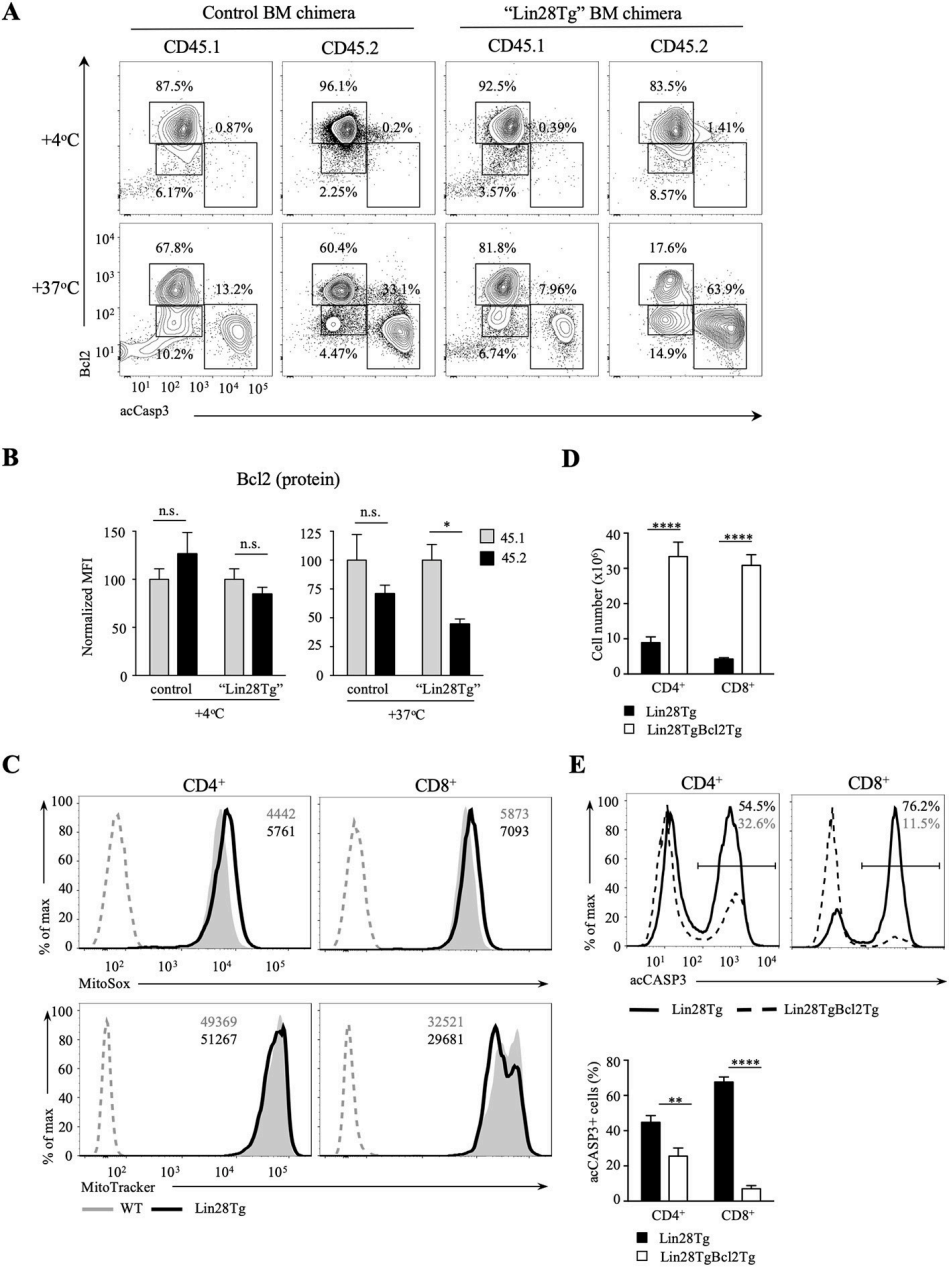
Bcl2 rescues cell death in let-7-deficient T cells. **(A)** Intracellular staining for Bcl2 and active Caspase-3 in CD8 lymph node T cells from control and “Lin28Tg” mixed BM chimeras cultured for 18 h in media. **(B)** MFI of Bcl2 in acCasp3 negative populations from CD8 lymph node T cells from control (*n* = 3) and “Lin28Tg” (*n* = 3) mixed BM chimeras cultured for 18 h in media. **(C)** Staining of fresh CD4 and CD8 lymph node T cells from wild type and Lin28Tg mice for mtROS (top) and mitochondria (bottom). MFIs of MitoSox and MitoTracker are shown. Dashed histogram shows unstained control. **(D)** Total number of CD4 and CD8 lymph node T cells recovered from Lin28Tg (*n* = 5) and Lin28TgBcl2Tg (*n* = 2) mice. **(E)** Intracellular staining for active Caspase-3 (top) and frequency of acCasp3^+^ population (bottom) in CD4 and CD8 lymph node T cells from Lin28Tg and Lin28TgBcl2Tg mice. Data are from three **(A–C)** and two **(D,E)** independent staining experiments. Data in **(B–D)** are displayed as mean ± SEM; n.s., not significant (*p* > 0.05), *p < 0.05, **p < 0.01, ****p < 0.0001.

Next, we tested whether intrinsic, or mitochondrial, apoptosis is involved in Lin28Tg T cell death. The intrinsic pathway is triggered by various stress signals and is regulated by Bcl-2 (B-cell lymphoma-2) family members that consist of two groups: pro-survival factors, such as Bcl-2, Bcl-XL, and Mcl1 and pro-apoptotic factors, that include the actual executioners of apoptosis, such as Bax and Bak, and BH3-only proteins, such as Bim, Noxa, Puma, and others (44). Anti-apoptotic and pro-apoptotic proteins interact with each other to regulate apoptosis, where both the balance between the Bcl-2 family members and the specificity of interactions are important for cell survival (45). In T cells, Bim has been shown to be the major player in regulating cell homeostasis (46). In particular, the Bim/Bcl-2 interaction is critical to control survival of naïve T cells (47). Moreover, it has been demonstrated previously that reduction in Bcl2 levels in activated T cells releases Bim that can then trigger cell death (48). Indeed, as shown in Figure S3E, Bim deficiency partially restored survival of Lin28Tg T cells during *in vitro* culture, indicating that mitochondria-mediated apoptosis may be responsible for the death of let-7-deficient T cells. However, protein expression of Bim was not different between WT and Lin28Tg T cells (Figure S3F), which suggested that it might be Bcl2 expression that is reduced in Lin28Tg T cells. While *Bcl2* mRNA level was significantly lower in Lin28Tg T cells (Figure 4A), protein expression showed little difference. The absence of Bcl2-low cells in the pool of *ex vivo* isolated lymphocytes could be due to a quick elimination of dead cells *in vivo*. *In vitro* apoptosis assay demonstrated that Lin28Tg T cells had a dramatic decrease in the frequency of the Bcl2-positive population among live (acCasp3-negative) CD4^+^ and CD8^+^ T cells in comparison to wild type and Let7Tg controls (Figure 4B, for quantification see Figure S3G). In addition, Bcl2 protein expression was significantly reduced in live Lin28Tg T cells and increased in Let7Tg T cells (Figure S3H). Again, to demonstrate that Bcl2 suppression is cell-intrinsic, we performed intracellular staining for Bcl2 and acCasp3 on the cultured LN cells from mixed BM chimeras (Figure 3C). Only Lin28Tg T cells (CD45.2^+^) from Lin28Tg chimera cultured at 37°C demonstrated profound reduction in the frequency of the Bcl2-positive population (Figure 5A) and Bcl2 MFI among acCasp3-negative cells (Figure 5B) in comparison to control cells at 4°C. On the contrary, wild type cells (CD45.1^+^) from the same chimera at 37°C showed very little difference when compared to 4°C culture.

To obtain more evidence of mitochondrial involvement in the impaired survival of let-7-deficient T cells, we analyzed the level of mitochondrial reactive oxygen species (mtROS) in freshly isolated Lin28Tg and wild type T cells, since increase in mtROS could be an indication of stressed mitochondria. Production of mtROS was higher in Lin28Tg CD4^+^ and CD8^+^ T cells in comparison to wild type controls (Figure 5C, top panels). Moreover, increased levels of mtROS was accompanied by lower mitochondrial mass in Lin28Tg CD8^+^ T cells but not CD4^+^ T cells (Figure 5C, bottom panels) which is in line with the more profound apoptosis observed in CD8^+^ T cells throughout the study. Collectively, these data strongly suggest that the inability of naïve let-7-deficient T cells to sustain Bcl2 levels results in mitochondrial apoptosis.

To validate that reduced Bcl2 expression is responsible for the impaired survival of let-7-deficient T cells *in vivo*, double transgenic Lin28TgBcl2Tg mice were generated. Indeed, the total T cell numbers were significantly increased in the lymph nodes of Lin28TgBcl2Tg mice (Figure 5D), while the frequency of acCasp3-positive LN T cells in an *in vitro* apoptosis assay was dramatically reduced (Figure 5E) demonstrating that re-expression of Bcl2 in Lin28Tg T cells completely rescued the survival defect.

### IL-7 Signaling Is Not Affected in Let-7-Deficient T Cells

**Figure 6:**
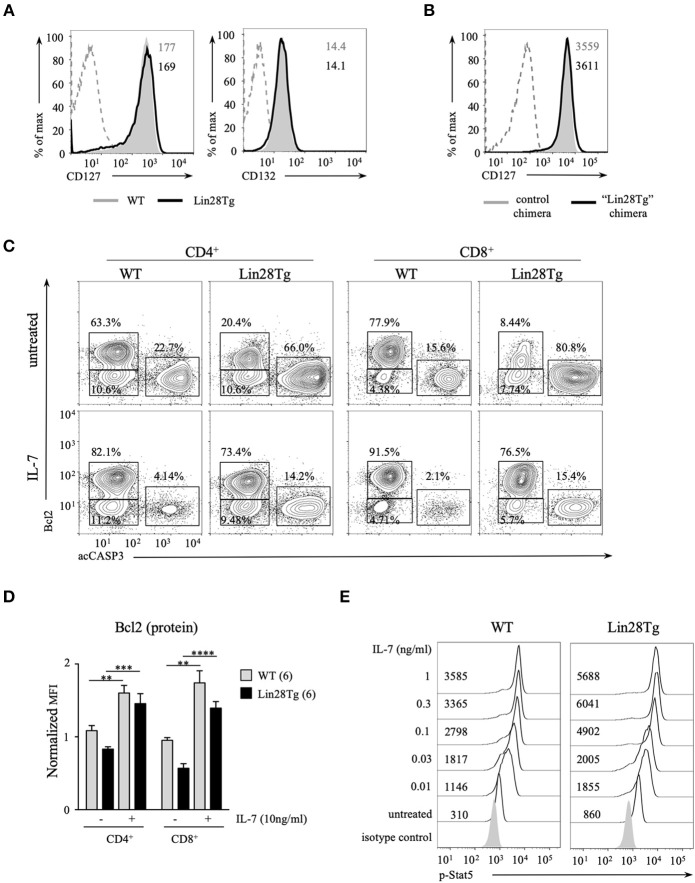
IL-7 receptor signaling is normal in let-7-deficient T cells. **(A)** Surface expression of CD127 and CD132 on CD8 T cells from lymph nodes of P14+Rag2KO wild type and Lin28Tg mice. MFIs of CD127 and CD132 are shown. Dashed histogram shows isotype control. **(B)** Surface expression of CD127 on CD8 T cells from lymph nodes of control and “Lin28Tg” mixed BM chimeras. MFIs of CD127 are shown. Dashed histogram shows isotype control. **(C)** Intracellular staining for active Bcl2 and active Caspase-3 in CD4 and CD8 lymph node T cells from wild type and Lin28Tg mice cultured for 18 h in the presence of 10 ng/ml IL-7. **(D)** MFI of Bcl2 in acCasp3 negative populations in CD4 and CD8 lymph node T cells from **(C)** normalized to +4°C cultures. **(E)** Phospho-Stat5 staining in CD8 T cells from lymph nodes of P14+Rag2KO wild type and Lin28Tg mice cultured with indicated concentrations of IL-7 for 30 min. MFIs of p-Stat5 are shown. Data in **(A,E)** are from two independent staining experiments. Data in **(B,C)** are representative of three independent staining experiments. Data in D are displayed as mean ± SEM; **p < 0.01, ***p < 0.001, ****p < 0.0001.

Given that Bcl2 expression in naïve T cells is mainly regulated by IL-7, we sought to determine whether IL-7 signaling is impaired in Lin28Tg T cells. Surface expression of both CD127, IL-7 receptor alpha-chain, and CD132, common gamma-chain, was unchanged on P14^+^Lin28TgRag2KO CD8^+^ T cells as compared to P14^+^Rag2KO (Figure 6A). In addition, CD8^+^ T cells isolated from control and Lin28Tg mixed BM chimeras also expressed similar levels of CD127 (Figure 6B). IL-7 rescued both survival of Lin28Tg T cells and Bcl2 levels in an *in vitro* apoptosis assay, when added at the concentration of 10 ng/ml (Figures 6C,D). We next tested whether more physiological amounts of IL-7 could also protect Lin28Tg T cells from apoptosis. Indeed, even at the lowest concentration of IL-7 (0.01 ng/ml), the frequency of acCasp3-positive cells started to decrease and Bcl2 expression began to go up in Lin28Tg T cells (Figures S4A–C). Moreover, phosphorylation of Stat5, the transcription factor downstream of the IL-7 receptor, was even more profound in Lin28Tg T cells than in wild type counterparts, and increased with increasing concentrations of IL-7 (Figure 6E). Altogether, these results demonstrate that signaling from IL-7 receptor is not affected in naïve let-7-deficient T cells. From our previous work we know that the TCR signaling pathway, another component of naïve T cell homeostasis, is not impaired in Lin28Tg T cells either, as they can be differentiated into potent functional effector CTLs (28). Therefore, neither IL-7 nor TCR signaling can account for the survival defect of let-7-deficient T cells. Moreover, intact responses to the main homeostatic signals explain the fact that T cells are reduced but still present in the periphery of Lin28Tg mice.

In conclusion, our data demonstrate that let-7-deficient T cells have suboptimal levels of Bcl2, which in turn leads to increased mitochondrial apoptosis. This clearly indicates that the let-7 miRNA family is an essential player in the regulation of naïve T cell homeostasis. Further investigation is required to determine the specific let-7-dependent mechanisms that are responsible for the survival and quiescence of naïve T cells.

## Discussion

Maintenance of the naïve T cell pool has been a topic of intensive research for many years and significant progress has been made in elucidating the mechanisms that control naïve T cell homeostasis, which includes both the long-term persistence of T cells and maintenance of their quiescence. We have previously shown that naïve CD8^+^ T cells express high levels of let-7 miRNAs and that let-7-deficient naïve T cells have elevated expression of activation markers, CD44 and CD122, a higher frequency of Ki67-positive T cells and demonstrate increased rates of homeostatic proliferation, all of which is indicative of the loss of their quiescent state (28). In this report, we show that survival, another essential component of peripheral naïve T cell homeostasis, is dramatically affected by the absence of let-7 miRNAs. Lin28Tg mice with T-cell specific expression of the Lin28 protein that inhibits let-7 maturation in T cells, demonstrated severe peripheral T cell lymphopenia due to apoptosis of naïve T cells. Lymphopenia in Lin28Tg mice has been reported before (34), however, it wasn't linked specifically to let-7 deficiency and the underlying mechanisms remained unexplored. We found that unrelated functions of Lin28 protein could not account for impaired survival because the overexpression of let-7Tg reversed the phenotype and protected T cells from apoptosis. Furthermore, using a mixed BM chimera approach, we showed that the survival defect of let-7-defecient T cells is cell intrinsic, since reconstitution of lethally irradiated C57BL/6 host mice resulted in the expansion of wild type but not Lin28Tg T cells.

As mentioned earlier, Lin28Tg T cell pool consists of about 50% of memory-like T cells with upregulated expression of CD44 and CD122. Moreover, we have shown previously that Lin28Tg T cells are Ki67-positive and are actively cycling (28). An interesting question is whether the activated phenotype of let-7-deficient T cells is related to excessive death of these cells. Naïve T cells undergo homeostatic proliferation both under experimentally induced lymphopenic conditions and during naturally occurring lymphopenia, such as in neonatal and elderly organisms (49). In response to homeostatic proliferation naïve T cells acquire a memory-like phenotype with increased expression of some, but not all, memory markers, including CD44 and CD122 (2, 50). Given that Lin28Tg mice are lymphopenic, it is not surprising that let-7-deficient T cells attempt to fill the empty niche and thus possess the features of homeostatically expanding cells. However, analysis of the activation status of T cells from our mixed BM chimeras, demonstrated that in comparison to wild type T cells that appeared naïve, Lin28Tg T cells retained their memory-like phenotype and Ki-67 expression (data not shown), allowing us to conclude that the activated phenotype of T cells in the absence of let-7 miRNAs is cell-intrinsic.

Long-term maintenance of the peripheral naïve T cell pool depends on both TCR and IL-7 receptor engagement (1–3). The main function of IL-7 in supporting T cell survival is the regulation of the balance between Bcl2 family members. Specifically, the anti-apoptotic proteins Bcl2 and Mcl1 are induced by IL-7, while pro-apoptotic factors Bid, Bad and Bim are inhibited (51). The role of TCR signaling in this process was not entirely clear until recently, when it was shown that continuous IL-7 signaling in CD8^+^ T cells leads to cell death, revealing thus that the purpose of TCR engagement is to temporarily block IL-7 receptor transduction (52). We showed that let-7-deficient T cells express suboptimal levels of Bcl2 which led us to hypothesize that IL-7 signaling in these cells might be compromised. Surprisingly, we found no evidence of that, as surface expression of the IL-7 receptor was similar in Lin28Tg and wild type T cells, Stat5 phosphorylation upon IL-7 receptor ligation was even increased in Lin28Tg T cells, and IL-7 rescued T cell survival in the *in vitro* apoptosis assay. Memory cells are known to express higher levels of Bcl2 (53) and thus normally exhibit a better survival. However, in spite of the high frequency of memory-like T cells in Lin28Tg mice, majority of Lin28Tg T cells loose Bcl2 expression in an *in vitro* apoptosis assay and die, which suggests that both naïve and memory-like let-7-deficient T cells have a survival defect.

Interestingly, we have shown previously that thymocyte numbers and frequencies were unaffected by the absence of let-7 miRNAs in Lin28Tg mice (30). Two factors might compensate for the Bcl2 loss in Lin28Tg T cells in the thymus: (i) Bcl-XL, which supports survival of double positive thymocytes and is expressed at higher levels on single positive thymocytes in comparison to peripheral T cells (54) and (ii) IL-4, that is produced at high levels in the thymus (55) and is also known to promote survival.

Expression of Bcl2 family members is regulated on multiple levels, including post-transcriptional and post-translational. Several RNA binding proteins have been demonstrated to control mRNA splicing, stability and subcellular localization of Bcl2 family proteins (56). For example, HuR, nucleolin, AUF1, and ZFP36L1 are among a few that are involved in stabilization/destabilization of Bcl2 mRNA (57–60). In addition, various miRNAs have been validated to target Bcl2 and other members of the family. Post-translational modifications of proteins can dramatically affect their half-life, localization, interactions and activity. Multiple modifications have been shown to be essential for the function of Bcl2 family proteins, including phosphorylation, ubiquitination, carbonylation, and S-nitrosylation (61). We speculate that stability of Bcl2 either on the post-transcriptional or post-translational level might be compromised in the absence of let-7 miRNAs. In the future, it would be very interesting to determine how let-7 affects the signaling pathways that control the stability of Bcl2 message and/or protein.

## Materials and Methods

### Mice

The following mice were purchased from the Jackson Laboratory: C57BL/6J (stock 000664), B6.SJL-*Ptprc*^*a*^*Pepc*^*b*^/BoyJ (CD45.1, stock 002014), B6(Cg)- *Rag2*^*Tm*1.1*Cgn*^/J (*Rag2*^−/−^, stock 008449), B6.Cg-*Col1a1*^*tm*3(*tetO*−*Mirlet*7*g*/*Mir*21)*Gqda*^/J (let-7g, stock 023912) and B6.Cg-*Gt(ROSA)26 Sor*^*tm*1(*rtT*^A^^*^M2)*Jae*^/J (M2rtTA, stock 006965). Let-7g and M2rtTA mice were crossed to generate Let7Tg mice. Mice with the Lin28B transgene (Lin28Tg) (30), B6; D2-Tg(TcrLCMV)327Sdz/JDvs/J (P14) mice and CD4-Cre*Dcr*^fl/fl^ (62) mice were a generous gift from Alfred Singer (NCI, NIH). All animal experiments were performed in accordance with the guidelines of the US National Institutes of Health and the protocols approved by the IACUC of the University of Massachusetts.

### Doxycycline-Mediated Induction of Let-7 Transgene Expression

In the experiments that involved Let7Tg mice, all animals including controls were fed with 2 mg/mL doxycycline in drinking water supplemented with 10 mg/mL sucrose for 4 days prior to lymph node isolation to ensure maximal induction of let-7g expression. Doxycycline drinking water was replaced once over the course of 4 days. Then lymphocytes were cultured *in vitro* with 2 μg/mL doxycycline in culture media (see *in vitro* culture below).

### *In vivo* BrdU Labeling

Mice were injected i.p. with 1 mg BrdU in PBS, and subsequently fed with 0.8 mg/mL BrdU in drinking water supplemented with 2% sucrose for 4 days. BrdU water was kept in the dark to eliminate light-sensitivity effects of BrdU and was replaced daily. Incorporation of BrdU in T cells from lymph nodes was analyzed by flow cytometry.

### Generation of Mixed Bone Marrow Chimera

BM cells were prepared from WT (CD45.1^+^) and P14^+^Lin28TgRag2KO (CD45.2^+^) or P14^+^Rag2KO (CD45.2^+^) donor mice by flushing femurs and tibias. Cells were then depleted of mature T and B cells by incubation with anti-mouse CD4 (GK1.5) and CD8 (2.43) antibodies conjugated with rat IgG followed by incubation with anti-rat IgG magnetic beads and anti-mouse IgG magnetic beads (BioMag, Qiagen). Host C57BL/6J (CD45.1^+^) mice were lethally irradiated (900 Rad) and injected intravenously (tail vein) with a mixture of 6 × 10^6^ cells from WT (CD45.1^+^) and P14^+^Lin28TgRag2KO (CD45.2^+^) or WT (CD45.1^+^) and P14^+^Rag2KO (CD45.2^+^) donors mixed at 1:10 ratio. Chimeras were analyzed 8 weeks post-injection.

### Adoptive Transfer

Cells for adoptive transfer were obtained from lymph nodes of donor WT and Lin28Tg mice. Lymphocytes were electronically sorted for the purification of naïve T cells (CD44^lo^). Rag2KO host mice were injected intravenously (tail vein) with a mixture of 1 × 10^6^ cells from WT and Lin28Tg mice at 1:1 ratio. Host mice were analyzed every week starting 1 week post-injection for 4 weeks.

### Flow Cytometry

The following antibodies were used: CD4 FITC (clone GK1.5, BioLegend # 100406, PRID:AB_312691), CD8α PB (clone 5H10, Thermo Fisher Scientific # MCD0828, RRID:AB_10372364), PE Hamster Anti-Mouse Bcl-2 Set (Bcl-2 clone 3F11, American hamster IgG clone A19-3, BD Biosciences # 556537, PRID:AB_396457), cleaved caspase-3 (Cell Signaling # 9661, PRID:AB_2341188), CD44 FITC (clone IM7, BioLegend # 103006, PRID:AB_312957), CD122 biotin (clone TM-β1, BioLegend # 123206, PRID:AB_940609), BrdU Alexa Fluor 647 (clone 3D4, BioLegend # 364107, RRID:AB_2566451), Bim (BD Biosciences # 559685, PRID:AB_397305), Fas PE (clone SA367H8, BioLegend # 152607, PRID:AB_2632903), TNFR1 PE (clone 55R-286, BioLegend # 113003, PRID:AB_313532), CD45.2 FITC (clone 104, BD Pharmingen # 553772, RRID:AB_1727491), anti-Stat5 (pY694) (BD Biosciences # 612599, RRID:AB_399882), F(ab′)2-goat anti-rabbit IgG secondary Ab Alexa Fluor 647 (Thermo Fisher Scientific # A-21246, RRID:AB_2535814), Streptavidin Alexa Fluor 647 (BioLegend # 405237, RRID:AB_2336066). For intracellular staining, live cells were first stained for surface proteins and then fixed, permeabilized, and stained for intracellular proteins using the Foxp3 Transcription Factor Staining Buffer Set (eBioscience) according to the manufacturer's instructions. All flow cytometry data were acquired on a BD Fortessa and analyzed with FlowJo software (TreeStar).

### Mitochondria Labeling

To detect mtROS and to stain mitochondria, freshly isolated lymph node T cells were incubated with 5 μM of MitoSox Red (Molecular Probes) and 1 μM of MitoTracker Deep Red (Molecular Probes) for 30 min at 37°C in HBSS, washed one time, surface-stained for CD4 and CD8, and analyzed by Flow Cytometry.

### *In vitro* Culture

Lymph node cells were cultured in RPMI supplemented with 10% fetal bovine serum, 1% HEPES, 1% sodium pyruvate, 1% penicillin/streptomycin, 1% L-glutamine, 1% non-essential amino acids, 0.3% β-mercaptoethanol, 100 U/mL IL-2, 100 mg/mL gentamicin, and 2μg/mL doxycycline when necessary.

### Isolation of RNA and Quantitative PCR

RNA was isolated using Total RNA Purification Kit (Norgen bioteck) according to the manufacturer's instructions, and genomic DNA was removed using the DNA-free DNA removal kit (Ambion). mRNA- encoding cDNA was synthesized using SensiFAST cDNA Synthesis Kit (Bioline), while miRNA-encoding cDNA was synthesized using the Taqman MicroRNA Reverse Transcription kit (Thermo Fisher Scientific). SYBR Green quantitative PCR was performed using the SensiFAST SYBR Lo-Rox kit (Bioline). The following SYBR Green amplification primers (Integrated DNA Technologies) were used (forward, reverse): *Fas* 5′-TATCAAGGAGGCCCATTTTGC-3′, 5′-TGTTTCCACTTCTAAACCATGCT-3′; *FasL* 5′-GGTCTACTTACGATATCACAGAGGCCGTT-3′, 5′-CGGCCTCTGTGATATCGTAAGTAGACCCAC-3′; *Tnfrsf10b* 5′-CGGGCAGATCACTACACCC-3′, 5′-TGTTACTGGAACAAAGACAGCC-3′; *Tnfsf10* 5′-ATGGTGATTTGCATAGTGCTCC-3′, 5′-GCAAGCAGGGTCTGTTCAAGA-3′; *Tnfrsf1a* 5′-CCGGGAGAAGAGGGATAGCTT-3′, 5′-TCGGACAGTCACTCACCAAGT-3′; *Rpl13a* 5′-CGAGGCATGCTGCCCCACAA-3′, 5′-CGAGGCATGCTGCCCCACAA-3′, 5′-AGCAGGGACCACCATCCGCT-3′. The following Taqman MicroRNA assays were obtained from Thermo Fisher Scientific: hsa-let-7a—assay ID 000377, hsa-let-7b—assay ID 000378, hsa-let-7c—assay ID 000379, hsa-let-7d—assay ID 002283, hsa-let-7e—assay ID 002406, hsa-let-7f—assay ID 000382, hsa-let-7g—assay ID 002282, hsa-let-7i—assay ID 002221, U6—assay ID 001973. All quantitative PCR data were obtained using QuantStudio 6 Real-Time PCR machine and software (Applied Biosystems).

### Statistical Analysis

*P*-values were determined using a two-tailed Student's *t*-test.

## Data Availability

The raw data supporting the conclusions of this manuscript will be made available by the authors, without undue reservation, to any qualified researcher.

## Ethics Statement

This study was carried out in accordance with the guidelines of the US National Institutes of Health and the protocols were approved by the IACUC of the University of Massachusetts.

## Author Contributions

EP and LP designed the study, performed experiments, and interpreted the results. AW, CA, EF, EA, and MK performed experiments. EI provided technical support. EP drafted the manuscript. EP, AW, CA, and LP critically revised the manuscript.

### Conflict of Interest Statement

The authors declare that the research was conducted in the absence of any commercial or financial relationships that could be construed as a potential conflict of interest.
