# Supplementary figures and images for: Survival of Naïve T Cells Requires the Expression of Let-7 miRNAs
Source: Front Immunol. 2019 May 3;10:955. doi: 10.3389/fimmu.2019.00955 (PMC6509570; doi:10.3389/fimmu.2019.00955)

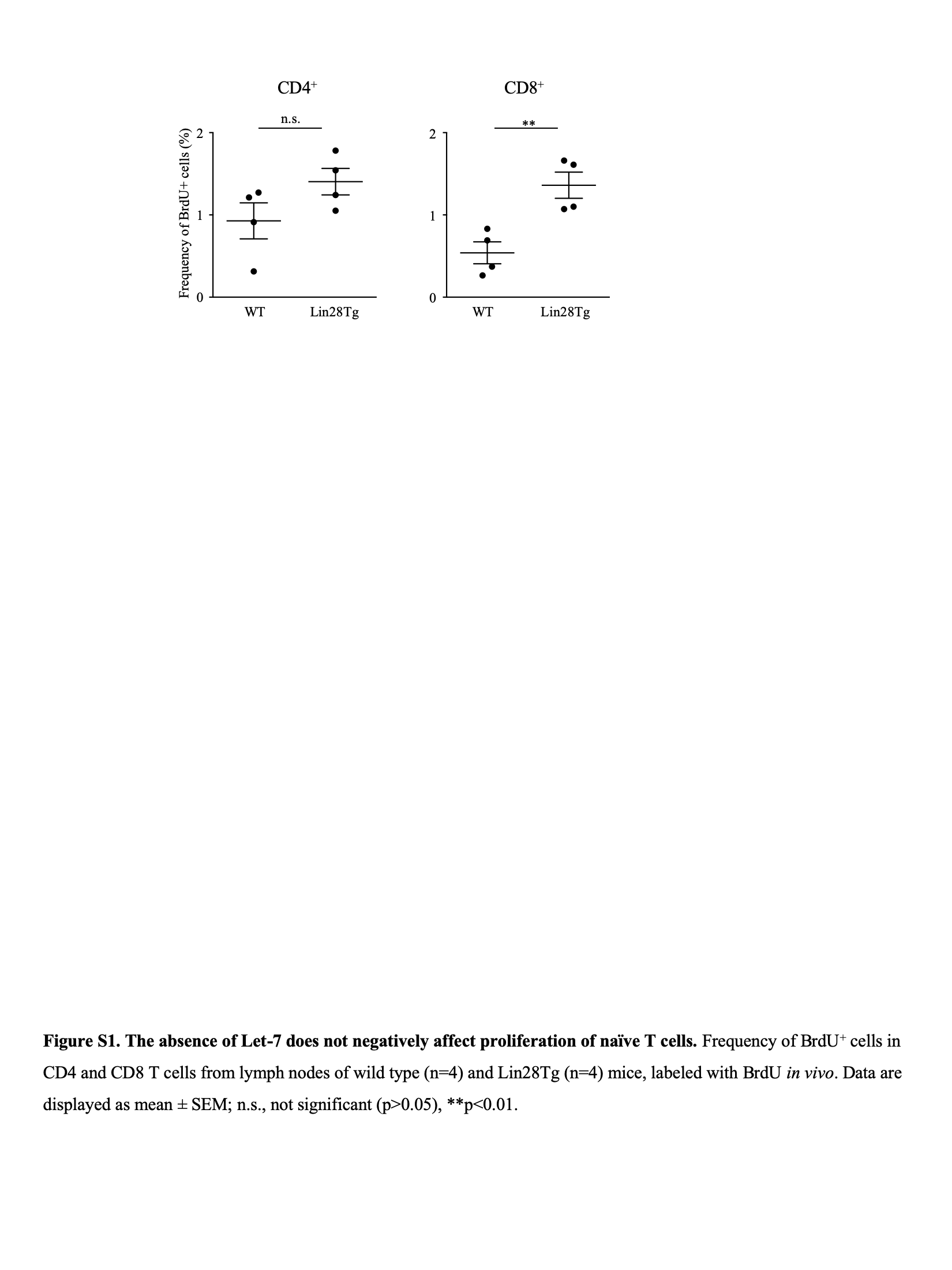

Supplement: Supplementary file 1 [file Image_1.TIFF]

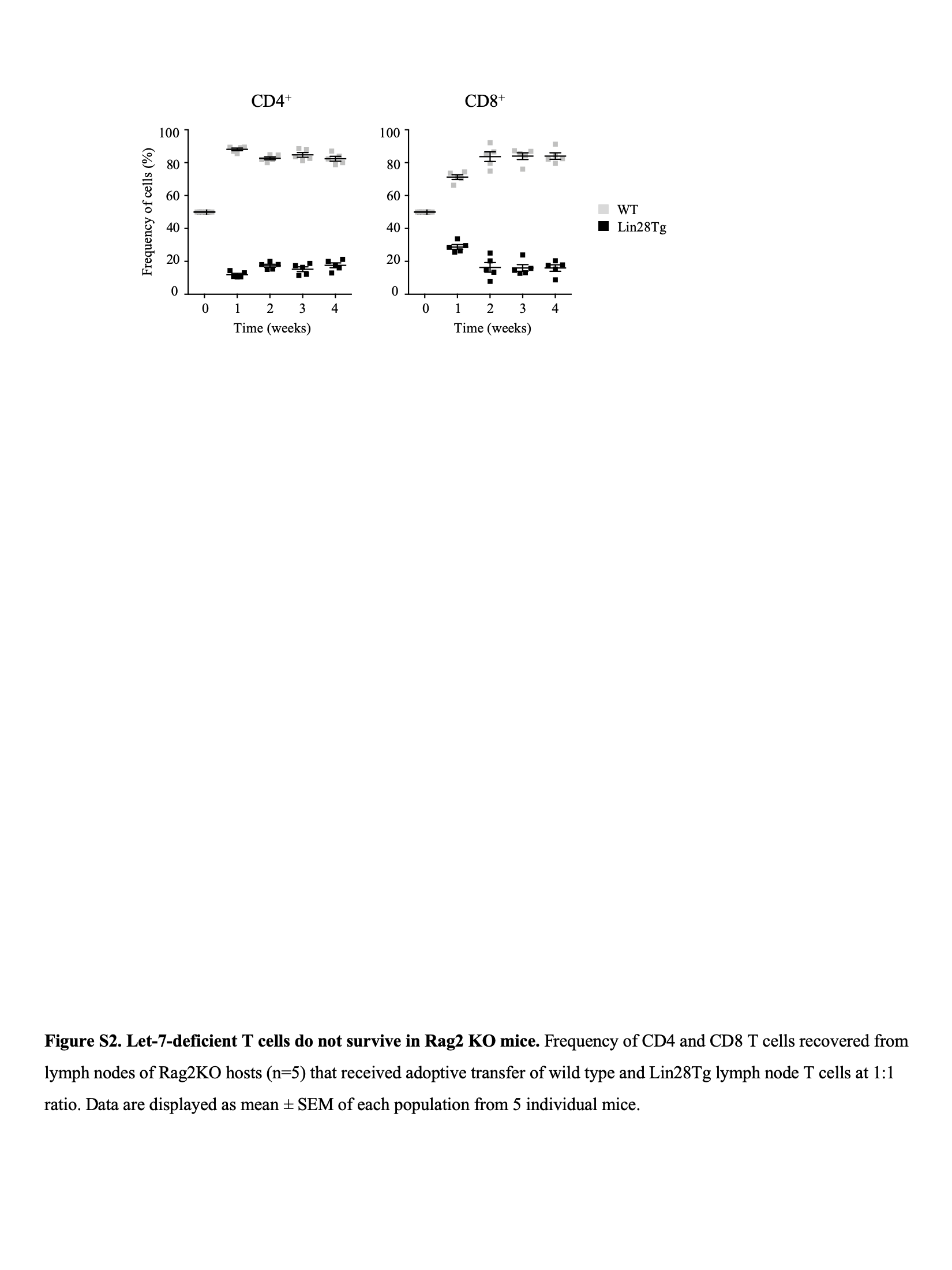

Supplement: Supplementary file 2 [file Image_2.TIFF]

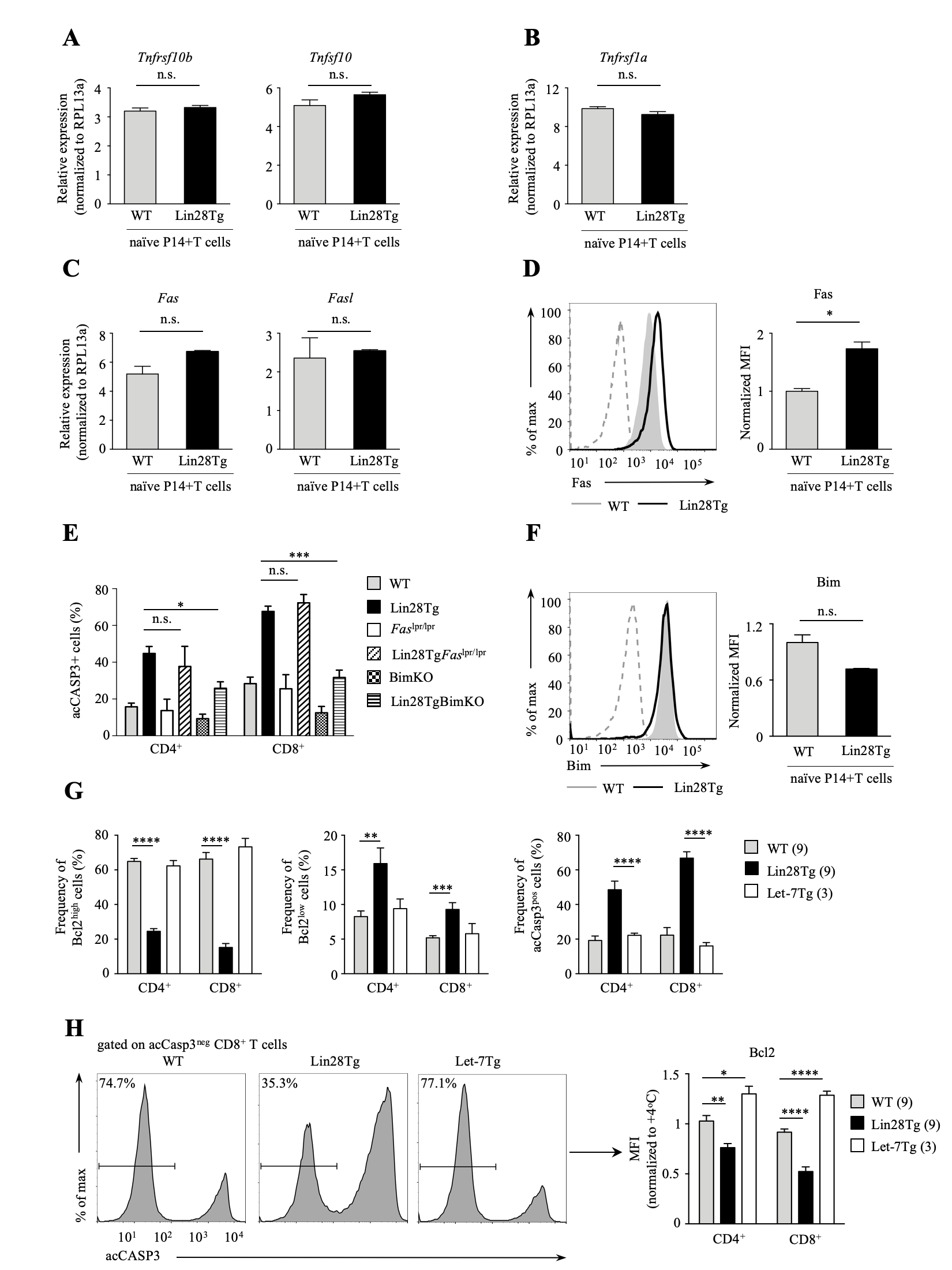

Supplement: Supplementary file 3 [file Image_3.TIFF]

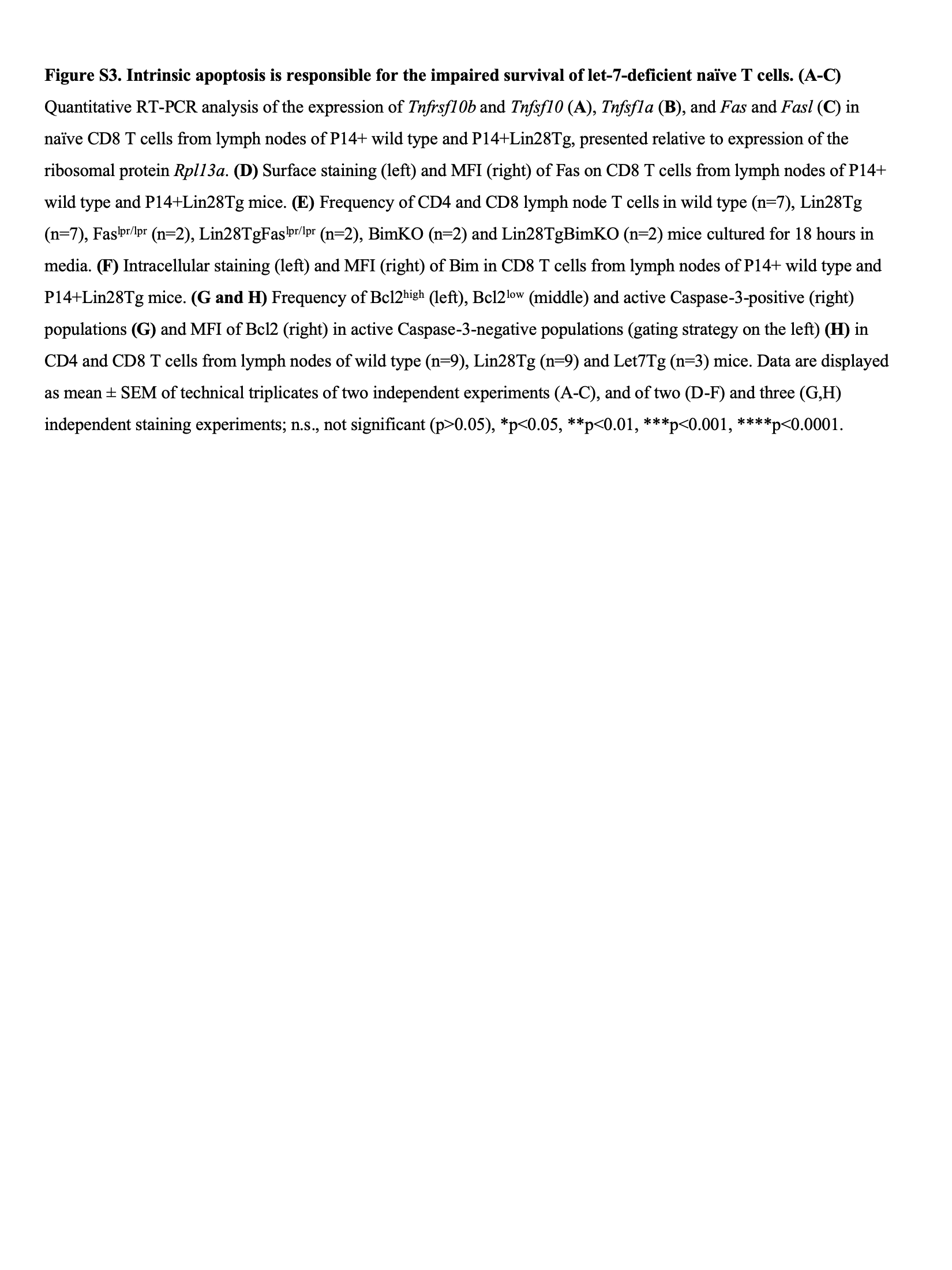

Supplement: Supplementary file 4 [file Image_4.TIFF]

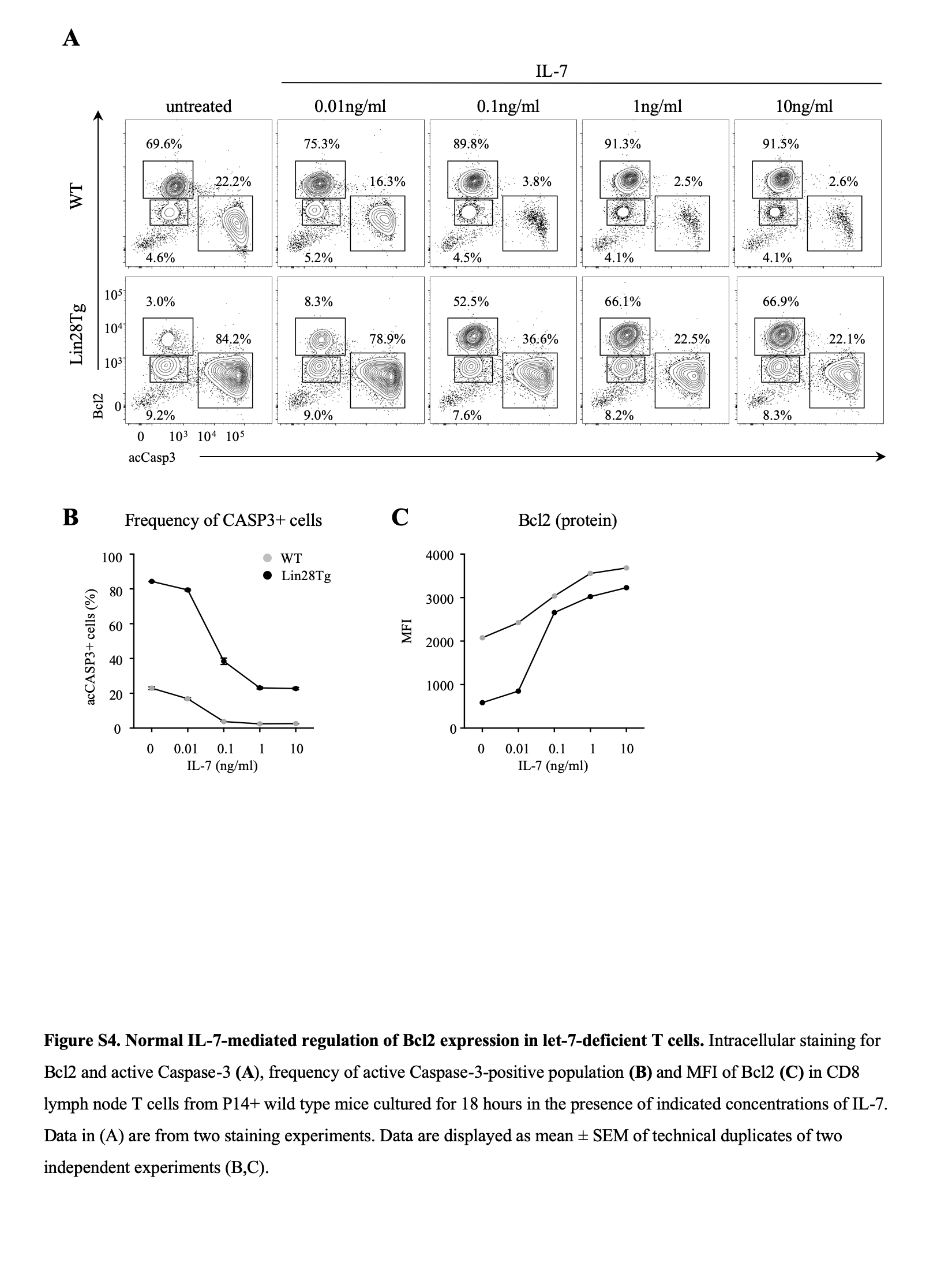

Supplement: Supplementary file 5 [file Image_5.TIFF]
